# Nursing Diploma of the University of Leeds

**Published:** 1921-06-25

**Authors:** 


### Nursing Diploma of the University of Leeds.

The following regulations for a diploma in nursing were adopted by the Council of the University of Leeds on May 25, 1921, and sanctioned by the Court of the University on June 15 :

1. Each candidate who satisfies the examiners shall receive a diploma and shall be styled a Diplomate in Nursing, University of Leeds.

2. Before presenting themselves for examination for the diploma, candidates must have completed four years' training in a general hospital recognised by the University for the purpose, and shall have received a certificate to this effect.

3. Candidates shall furnish evidence of having attained an adequate standard in general education satisfactory to the University.

4. The four years' hospital training shall include : (*a*) Practical instruction and tuition in the following : Ward nursing, medical, surgical, and special; the principles of surgical technique and operation service; bandaging and the preparation and use of splints and other appliances; invalid cookery; the feeding and management of infants and diseases of infancy and childhood; the principles of ward administration; elementary urine testing; preparation for autopsies. (*b*) Attendance on the following courses of lectures and lecture demonstrations : The principles and practice of nursing, twenty lectures; elementary anatomy and physiology, twenty lectures; elementary medicine, twelve lectures; elementary surgery, twelve lectures; elementary obstetrics and gynæcology, eight lectures; the management of infancy and childhood, six lectures; elementary hygiene, eight lectures. Candidates shall furnish certificates of such attendance and of having passed satisfactorily a class examination in each subject.

At least three months must be spent in attendance on courses in the University of Leeds (for this purpose lectures delivered in the Leeds General Infirmary shall be deemed to have been delivered in the University of Leeds). The number and character of the courses shall be determined in each case by the University.

5. Candidates must also attend a course of lectures in the University of Leeds on social economics or some other approved subject.

6. A nurse may be registered as a candidate for the examination at any time after she has been accepted for full hospital training, upon (*a*) payment of a registration fee of five guineas; (*b*) producing the necessary evidence of general education.

7. The examination may be taken at any time after lodging the certificate of completion of training (as defined in paragraph 2) upon (*a*) payment of a further fee of five guineas; (*b*) the production of the certificates of attendance upon the prescribed courses of lectures (paragraphs 4, 5, and 6).

8. The examination shall be held twice annually, and shall be by written papers, in practical work, *viva voce*. There shall be a written paper in each of the following subjects : The principles and practice of nursing; elementary medicine and surgery; elementary anatomy and physiology; elementary obstetrics and gynæcology; the care of infancy and childhood; and elementary hygiene.

